# Induction of ferroptosis promotes vascular smooth muscle cell phenotypic switching and aggravates neointimal hyperplasia in mice

**DOI:** 10.1186/s10020-022-00549-7

**Published:** 2022-10-03

**Authors:** Shunchi Zhang, Yanrou Bei, Yueling Huang, Yimin Huang, Lianjie Hou, Xi-Long Zheng, Yiming Xu, Shaoguo Wu, Xiaoyan Dai

**Affiliations:** 1 Department of Clinical Laboratory, Guangzhou Twelfth People’s Hospital, Guangzhou Municipal and Guangdong Provincial Key Laboratory of Molecular Target & Clinical Pharmacology, the NMPA and State Key Laboratory of Respiratory Disease, School of Pharmaceutical Sciences, Guangzhou Medical University, Guangzhou, 511436 Guangdong China; 2 Laboratory Medicine Center, Nanfang Hospital, Southern Medical University, Guangzhou, 510515 Guangdong China; 3 Experimental Animal Center, Guangzhou Medical University, Guangzhou, 511436 Guangdong China; 4 The Sixth Affiliated Hospital of Guangzhou Medical University, Qingyuan City People’s Hospital, Qingyuan, 511518 Guangdong China; 5 Department of Biochemistry & Molecular Biology, Cumming School of Medicine, University of Calgary, Calgary, AB T2N 4Z6 Canada; 6 School of Basic Medical Sciences, Guangzhou Medical University, Guangzhou, 511436 Guangdong China

**Keywords:** Ferroptosis, Neointimal hyperplasia, Vascular smooth muscle cells, Phenotypic switching, ROS

## Abstract

**Background:**

Stent implantation-induced neointima formation is a dominant culprit in coronary artery disease treatment failure after percutaneous coronary intervention. Ferroptosis, an iron-dependent regulated cell death, has been associated with various cardiovascular diseases. However, the effect of ferroptosis on neointima formation remains unclear.

**Methods:**

The mouse common right carotid arteries were ligated for 16 or 30 days, and ligated tissues were collected for further analyses. Primary rat vascular smooth muscle cells (VSMCs) were isolated from the media of aortas of Sprague-Dawley (SD) rats and used for in vitro cell culture experiments.

**Results:**

Ferroptosis was positively associated with neointima formation. In vivo, RAS-selective lethal 3 (RSL3), a ferroptosis activator, aggravated carotid artery ligation-induced neointima formation and promoted VSMC phenotypic conversion. In contrast, a ferroptosis inhibitor, ferrostatin-1 (Fer-1), showed the opposite effects in mice. In vitro, RSL3 promoted rat VSMC phenotypic switching from a contractile to a synthetic phenotype, evidenced by increased contractile markers (smooth muscle myosin heavy chain and calponin 1), and decreased synthetic marker osteopontin. The induction of ferroptosis by RSL3 was confirmed by the increased expression level of ferroptosis-associated gene *prostaglandin-endoperoxide synthase 2* (*Ptgs2*). The effect of RSL3 on rat VSMC phenotypic switching was abolished by Fer-1. Moreover, *N*-acetyl-l-cysteine (NAC), the reactive oxygen species inhibitor, counteracted the effect of RSL3 on the phenotypic conversion of rat VSMCs.

**Conclusions:**

Ferroptosis induces VSMC phenotypic switching and accelerates ligation-induced neointimal hyperplasia in mice. Our findings suggest inhibition of ferroptosis as an attractive strategy for limiting vascular restenosis.

## Background

Applying percutaneous transluminal coronary angioplasty (PTCA) in patients is a milestone event in the surgical treatment of coronary artery disease (CAD) (Gruntzig 1978; Gruntzig et al. 1979). However, mechanical injuries caused by resection of atherosclerotic plaques, angioplasty, and stent implantation contribute to vascular restenosis, which largely limits the long-term improvement of CAD (Weintraub 2007). Neointima formation is the leading pathological cause of vascular restenosis and results from excessive proliferation and phenotypic transformation of vascular smooth muscle cells (VSMCs) (Meyer and Bult 1997). Early studies have shown that endothelial stripping induces platelet and fibrin deposition immediately after injury and subsequently stimulates VSMC migration, proliferation, and extracellular matrix synthesis (Welt and Rogers 2002; Garas et al. 2001; Faxon et al. 1984). Jason et al. (1994) pointed out that a sudden and sharp increase in VSMC proliferation is necessary for neointimal formation. Unlike cardiomyocytes and skeletal muscle cells at the end of differentiation, VSMCs have high plasticity. This means they tend to switch from a contractile phenotype to a synthetic, inflammatory, and osteogenic phenotype in response to various stimuli (Campbell and Campbell 2012; Ailawadi et al. 2009). Lineage tracking experiments showed that VSMC phenotypic transition occurs in vivo (Albarran-Juarez et al. 2016; Feil et al. 2014). Importantly, increasing evidence has shown that phenotypic conversion of VSMCs from a quiescent contractile to a proliferative synthetic phenotype is crucial for neointima formation (Gomez and Owens 2012). Therefore, dissecting the mechanism of VSMC phenotypic modulation may help determine how to attenuate neointimal formation and restenosis, eventually improving the outcome of PTCA.

Ferroptosis is a newly identified iron-dependent form of regulated cell death (RCD), mainly characterized by the excessive accumulation of reactive oxygen species (ROS) and lipid peroxidation products (Stockwell et al. 2020). Ferroptosis is closely related to many biological processes, such as the metabolism of amino acids, iron, and polyunsaturated fatty acids (Yuan et al. 2016; Yang and Stockwell 2016; Wang et al. 2016; Gao et al. 2015), and the biosynthesis of glutathione, phospholipids, NADPH, and coenzyme Q10 (Shimada et al. 2016a, b). Glutathione peroxidase 4 (GPX4) plays a pivotal role in eliminating cellular lipid hydroperoxide and functions as a central negative regulator of ferroptosis (Zhang et al. 2021; Jia et al. 2020). In *ApoE*^*−/−*^ mice, transgenic overexpression of human GPX4 delays the development of atherosclerosis by reducing lipid peroxidation (Guo et al. 2008). In rats, a recent study showed that endothelial cell ferroptosis contributes to monocrotaline‑induced pulmonary hypertension (Xie et al. 2022). However, the role of ferroptosis in neointimal formation and restenosis remains unexplored.

Emerging evidence shows that high hydrostatic pressure results in ferroptosis and induces VSMC inflammation (Jin et al. 2022). In addition, cigarette smoke extract triggers ferroptosis in VSMCs (Sampilvanjil et al. 2020). Overexpression of GPX4 significantly decreases oxidized low-density lipoprotein (oxLDL)-induced proliferation of VSMCs (Brigelius-Flohe et al. 2000). Although several identified types of RCD, such as apoptosis (Ostriker et al. 2021), autophagy (Pi et al. 2021), and necrosis (Champagne et al. 1999), have been implicated in VSMC function regulation, the effect of ferroptosis on VSMC function has not been determined. Therefore, the present study aims to investigate the effect of ferroptosis on VSMC phenotypic transformation and physical injury-induced vascular neointimal hyperplasia in mice.

## Materials and methods

### Chemicals and reagents

RAS-selective lethal (RSL3, S8155) and ferrostatin-1 (Fer-1, S7243) were purchased from Selleck Chemicals (Shanghai, China) and dissolved in dimethyl sulfoxide (DMSO, V900090, Sigma-Aldrich). RSL3 and Fer-1 were prepared for intraperitoneal (i.p.) injection as follows: 1% RSL3 or Fer-1 + 30% PEG300 (S6704, Selleck) + 5% Tween 80 (S6702, Selleck) + 64% H_2_O. *N*-Acetyl-l-cysteine (NAC, A9165, Sigma-Aldrich) was used. An optimal cutting temperature compound (OCT, 14020108926) was obtained from Leica (Wetzlar, Germany).

### Animals

Animal experiments were approved by the Animal Research Ethic Committee of Guangzhou Medical University (Protocol number: 2019-061) and performed following the NIH Guide for the Care and Use of Laboratory Animals. Male 8-week-old C57BL/6J mice were purchased from Shanghai Model Organisms (Shanghai, China). All mice were fed a chow diet, given autonomous access to water and food, maintained in the specific pathogen-free (SPF) facility, and kept on a 12 h light–dark cycle. For ligation of the common right carotid artery, mice were anesthetized with isoflurane on a heated stage. All hair on the neck between the mandible and sternum was gently removed using a suitable amount of depilatory agent, and an incision was made in the middle of the neck to find the common right carotid artery, which was ligated with 6-0 silk suture. Mice were randomly assigned to the vehicle (i.p. injection of DMSO, daily), RSL3 (i.p. injection of 10 mg/kg, daily), or Fer-1 (i.p. injection of 1 mg/kg, daily) groups. Cross-sections of carotid arteries were stained with hematoxylin and eosin (H&E). Intimal and medial areas were measured using Image J software (National Institutes of Health, Bethesda, MD, USA).

### Cell culture

Primary cultures of VSMCs were obtained from the media of aortas of SPF Sprague-Dawley (SD) rats (body weight 150–180 g) by tissue explant method, as described previously (Chi et al. 2017). The rat VSMCs were cultured in Dulbecco’s modified Eagle’s medium (DMEM, C11995500BT, Gibco) containing 10% fetal bovine serum (FBS, 10270-106, Gibco), and 100 U/ml penicillin/streptomycin (15140-122, Gibco). Primary rat VSMCs from 4 to 7 passages were used for the experiments. Cells with ~ 70% confluency were treated.

### Western blotting

Cells were washed twice with PBS after treatment. 50 μl pre-cooled RIPA lysis buffer (FD008, FUDE) was added to cells and placed on ice for 10 min. The protein extraction was collected using a cell scraper into an Eppendorf (EP) tube, followed by centrifugation at 13,000*g* and 4 °C. The supernatant was taken and transferred into a new EP tube. After the protein concentration was determined using the bicinchoninic acid (BCA, P0009, Beyotime) method, 4× loading buffer was added to the samples, mixed evenly, and heated at 100 °C for 10 min. 30 μg protein was separated by 10% SDS-PAGE gels and transferred to nitrocellulose membranes (66485, PALL). After blocking with 5% skim milk for 1 h at room temperature, the membranes were incubated with primary antibodies at 4 °C overnight. Membranes were then washed and incubated with HRP-conjugated secondary antibodies, washed again, and visualized with HRP chemiluminescent substrates (WBKLS0500, Millipore). Two pre-stained protein markers (abs923, absin; MP102-02, Vazyme) were used. Primary antibodies were as follows: smooth muscle myosin heavy chain (SM-MHC, ab53219, Abcam), calponin 1 (ABT129, Millipore), osteopontin (OPN, ab11503, Abcam), and β-actin (sc-47778, Santa Cruz). β-actin was used as the internal control. Quantification of band densitometry was done using AI 600 (GE, MA, USA).

### Quantitative RT-PCR (qRT-PCR)

Total RNA from rat VSMCs was extracted using TRIzol reagent (21101, AG). The purity and concentration of the RNA were determined by Thermo Scientific NanoDrop One. A reverse transcription kit (11706, AG) was applied to synthesize cDNA, and SYBR Green real-time PCR premix kit (11701, AG) was used to measure the mRNA levels. qRT-PCR was performed on a LightCycler^®^ 480 Instrument II (Roche Applied Science, CA, USA). The following primers were used (5′-3′): *Myh11 (SM-MHC)* forward: ATCACGGGGGAGCTGGAAAA; *Myh11 (SM-MHC)* reverse: AATGAACTTGCCAAAGCGGG; *Acta2 (α-SMA)* forward: CATCCGACCTTGCTAACGGA; *Acta2 (α-SMA)* reverse: AGAGTCCAGCACAATACCAGT; *Cnn1 (Calponin 1)* forward: GCCCAGAAATACGACCACCA; *Cnn1 (Calponin 1)* reverse: TGGAGCTTGTTGATAAATTCGCA; *Spp1 (OPN)* forward: CAGTCGATGTCCCTGACGG; *Spp1 (OPN)* reverse: GTTGCTGTCCTGATCAGAGG; *Ptgs2 (COX-2)* forward: TCCTCCTGTGGCTGATGACT; *Ptgs2 (COX-2)* reverse: CGGGATGAACTCTCTCCTCA; *Gpx4* forward: CCATTCCCGAGCCTTTCAAC; *Gpx4* reverse: CGGTTTTGCCTCATTGCGAG; and *Gapdh* forward: ATTGTCAGCAATGCATCCTG; *Gapdh* reverse: ATGGACTGTGGTCATGAGCC.

### Immunofluorescence

Immunofluorescence staining was performed after fixation in 4% (v/v) formaldehyde (G1101, Servicebio) and permeabilization with 0.5% Triton X-100 in PBS, supplemented with 10% goat serum for 1 h. Samples were incubated with primary antibodies for cyclooxygenase-2 (COX-2, ab15191, Abcam) and α-smooth muscle actin (α-SMA, ab5694, Abcam) at 4 °C overnight and then incubated with Goat anti-Rabbit Alexa Fluor Plus 555 (1:1000) secondary antibody (A32732, Invitrogen) for 1 h at room temperature. After washing three times in PBS, samples were counterstained with 4′,6-diamidino-2-phenylindole (DAPI, D1306, Invitrogen) for 15 min, and fluorescence was preserved using Fluoromount-G (0100-01, SouthernBiotech). Images were captured by a Nikon A1R confocal microscope (Tokyo, Japan). Mean fluorescence intensity (MFI, AU) and relative fluorescence intensity was calculated using Image J software.

### Perls’ Prussian blue staining

Perls’ Prussian blue staining was used to analyze iron deposition in neointima. The cross-sections of ligated carotid arteries were fixed with 4% (v/v) formaldehyde (G1101, Servicebio) and washed with PBS 3 times. Sections were incubated in Perl’s solution (G1422, Solarbio) for 30 min, and deionized water was used to rinse three times. After incubation with Nuclear Fast Red (G1422, Solarbio) for 7.5 min, the sections were set in 75%, 85%, 95%, and 100% ethanol for rapid gradient dehydration. Iron^+^ cells were counted at ×200 magnification.

### Statistical analysis

Statistical analyses were conducted using Graph Pad Prism 8 (Graph Pad Software). Data were presented as the mean ± SEM. Two groups were compared using a two-tailed unpaired Student’s *t*-test. Multiple groups were analyzed using one-way ANOVA followed by Tukey’s multiple comparisons test. *P* < 0.05 was considered statistically significant.

## Results

### Ferroptosis is positively associated with neointimal hyperplasia

**Fig. 1:**
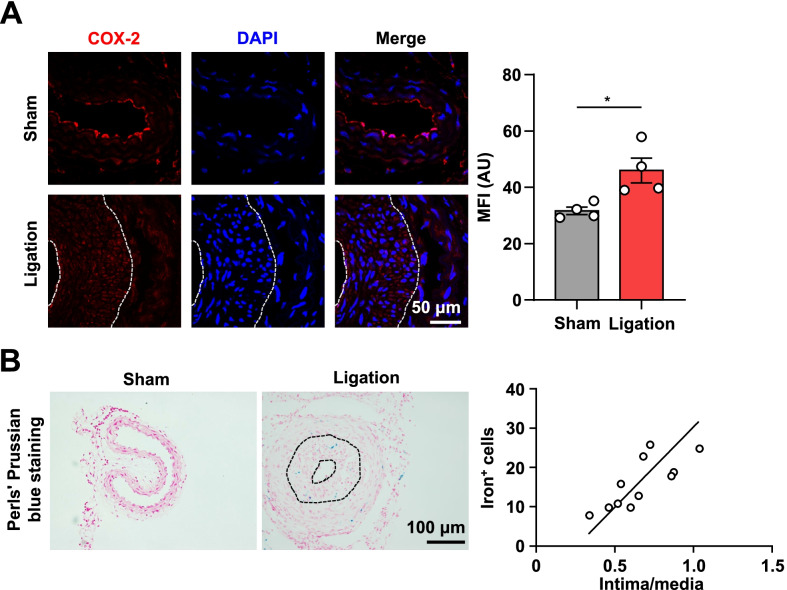
Ferroptosis is positively associated with neointima formation. **A** The presentative images and quantification of COX-2 mean fluorescence intensity (MFI) in mouse carotid artery after 4 weeks of ligation or not (*n* = 4 per group). Scale bar, 50 μm. **B** Perls’ Prussian blue staining of mouse carotid artery after 4 weeks of ligation. The correlation of intima/media and iron^+^ cells in neointimal formation (*n* = 11). r = 0.771, *P* = 0.0054. Scale bar, 100 μm. Data are presented as mean ± SEM, *P* values calculated by a two-tailed unpaired Student’s *t*-test. *P < 0.05

To study whether ferroptosis correlates with neointimal formation, we established a widely used vascular remodeling model through ligating the common right carotid artery of C57BL/6J mice. After 28 days of ligation, we measured ferroptosis in the neointima of the carotid artery section by immunofluorescence analysis of COX-2, a suitable marker of ferroptosis (Yang et al. 2014; Zhang et al. 2019). Our results showed that COX-2 is significantly increased in the neointima (Fig. 1A). However, COX-2 was dominantly expressed in vascular endothelial cells of the normal carotid artery without neointima formation (Fig. 1A). Moreover, we performed Perls’ Prussian blue staining to detect iron deposition in the neointima and calculated the Pearson correlation coefficient to assess correlations between the number of iron^+^ cells and the intima-to-media ratio of neointima. As shown in Fig. 1B, the number of iron^+^ cells was positively associated with the intima-to-media ratio of neointima (r = 0.771, *P* = 0.0054). Together, these findings suggest that ferroptosis may play a causal role in neointimal hyperplasia.

### A ferroptosis inducer, RSL3, accelerates neointimal hyperplasia after carotid artery ligation in mice

**Fig. 2:**
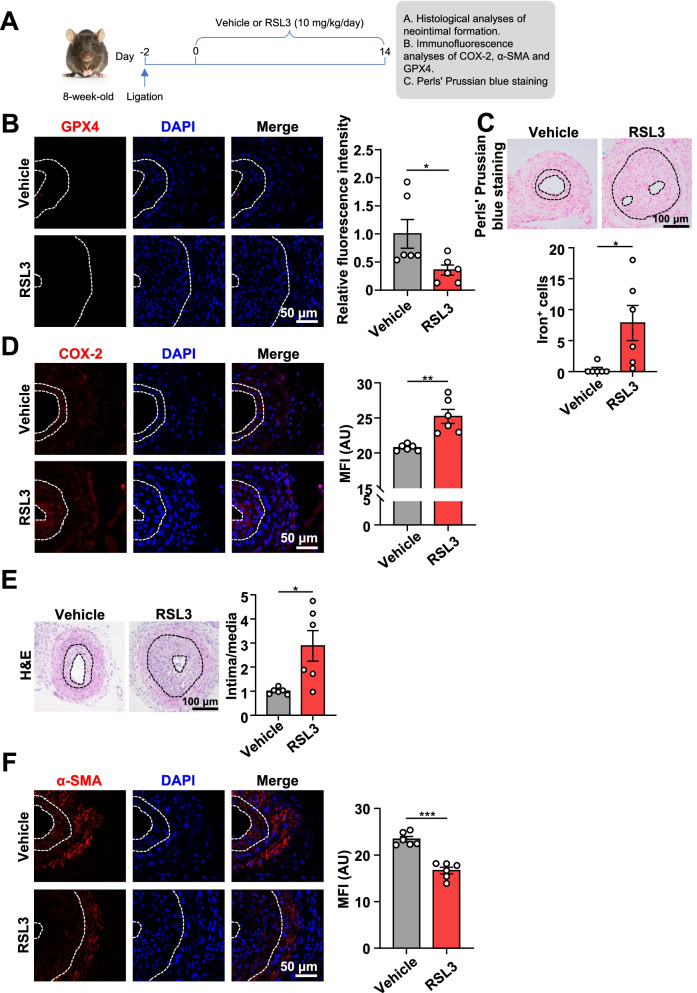
RSL3, a ferroptosis activator, accelerates ligation-induced neointima formation in mouse carotid arteries. **A** An experimental protocol. Two days after ligation, C57BL/6J mice were intraperitoneally injected with vehicle or RSL3 (10 mg/kg/day) for 14 days. Then, the carotid arteries were collected for further analysis. **B** Immunofluorescent staining of GPX4 in mouse carotid artery (neointima outlined by white dashed line). Quantification of GPX4 relative fluorescence intensity (*n* = 6 per group). Scale bar, 50 μm. **C** Perl’s Prussian blue staining in mouse carotid artery (neointima outlined by black dashed line). The number of iron^+^ cells in neointima per field (*n* = 6 per group). Scale bar, 100 μm. **D** Immunofluorescent staining of COX-2 in mouse carotid artery (neointima outlined by white dashed line). Quantification of COX-2 MFI (*n* = 6 per group). Scale bar, 50 μm. **E** H&E staining of ligated carotid arteries from C57BL/6J mice treated with RSL3 or vehicle (neointima outlined by black dashed line). The intima/media ratio was calculated (*n* = 6 per group). Scale bar, 100 μm. **F** Immunofluorescent staining of α-SMA in mouse carotid artery (neointima outlined by white dashed line). Quantification of α-SMA MFI (*n* = 6 per group). Scale bar, 50 μm. Data are presented as mean ± SEM, *P* values calculated by a two-tailed unpaired Student’s *t*-test. *P < 0.05, **P < 0.01, ***P < 0.001

RSL3, a well-established ferroptosis inducer, binds to and inactivates its substrate GPX4 (Yang et al. 2014). To determine if ferroptosis plays a role in neointima hyperplasia, we treated C57BL/6J mice with either RSL3 or vehicle, as indicated in Fig. 2A. The inhibition of GPX4 was demonstrated by lower expression of GPX4 in the neointima of RSL3-treated mice compared to vehicle-treated mice (Fig. 2B). In addition, the induction of ferroptosis was confirmed by more iron^+^ cells and higher expression of COX-2 in the neointima of RSL3-treated mice compared to vehicle-treated mice through Perls’ Prussian blue and immunofluorescence staining (Fig. 2C, D). Notably, at 16 days after carotid artery ligation, RSL3 treatment significantly increased the intima-to-media ratio of ligated carotid arteries compared with vehicle, indicating that RSL3 contributed to the neointimal hyperplasia in response to the injury (Fig. 2E).

The transition of the VSMC phenotype from a contractile/differentiated to a synthetic/dedifferentiated state is a prerequisite for neointima formation following vascular injury. We next asked whether ferroptosis could induce the dedifferentiated phenotype of VSMCs in vivo and therefore promote neointima formation after carotid artery ligation. We performed immunofluorescence staining for α-SMA, a specific VSMC contractile protein, in the ligated carotid arteries. The results showed that the expression of α-SMA in the neointima of RSL3-treated mice was significantly lower than in vehicle control mice (Fig. 2F), indicating that the induction of ferroptosis induced VSMC dedifferentiation and consequently aggravated neointima hyperplasia.

These results demonstrate that ferroptosis inducer RSL3 promotes neointimal hyperplasia in response to carotid artery ligation in mice in vivo.

### A ferroptosis inhibitor, Fer-1, alleviates neointima formation

**Fig. 3:**
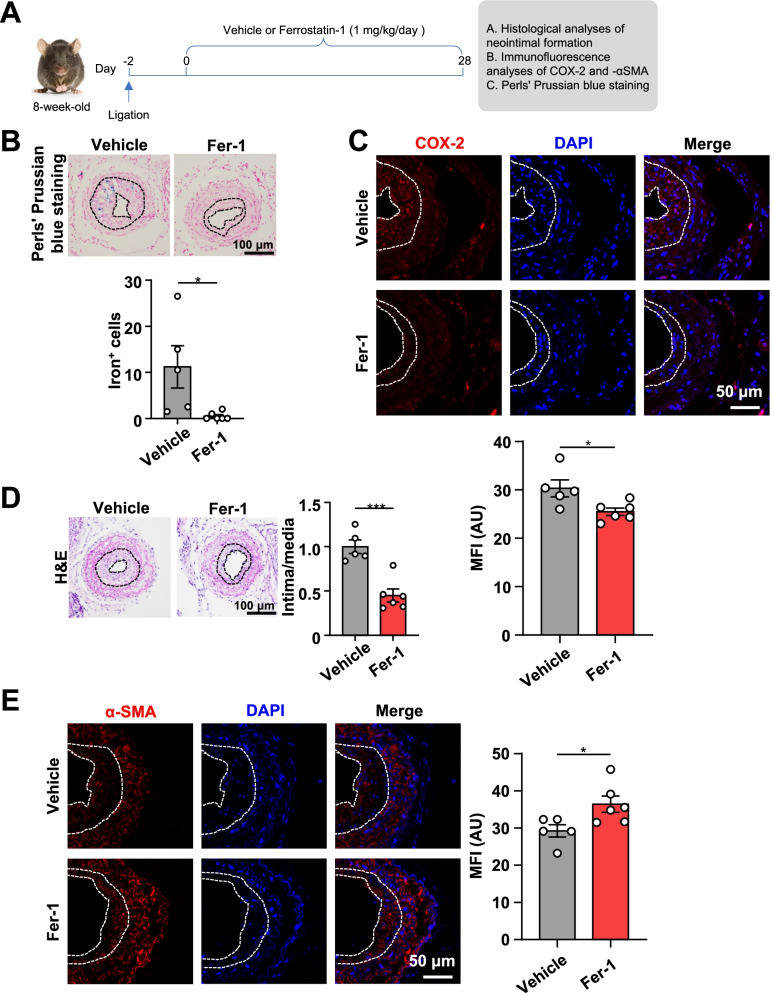
Fer-1, a ferroptosis inhibitor, limits ligation-induced neointima formation in mouse carotid arteries. **A** An experimental protocol. Two days after ligation, C57BL/6J mice were intraperitoneally injected with vehicle or Fer-1 (1 mg/kg/day) for 28 days. Then, the carotid arteries were collected for further analysis. **B** Perl’s Prussian blue staining in mouse carotid artery (neointima outlined by black dashed line). The number of iron^+^ cells in neointima per field (*n* = 5, vehicle and *n* = 6, Fer-1). Scale bar, 100 μm. **C** Immunofluorescent staining of COX-2 in mouse carotid artery (neointima outlined by white dashed line). Quantification of COX-2 MFI (*n* = 5, vehicle and *n* = 6, Fer-1). Scale bar, 50 μm. **D** H&E staining of ligated carotid arteries from C57BL/6J mice treated with Fer-1 or vehicle (neointima outlined by black dashed line). The intima/media ratio was calculated (*n* = 5, vehicle and *n* = 6, Fer-1). Scale bar, 100 μm. **E** Immunofluorescent staining of α-SMA in mouse carotid artery (neointima outlined by white dashed line). Quantification of α-SMA MFI (*n* = 5, vehicle and *n* = 6, Fer-1). Scale bar, 50 μm. Data are presented as mean ± SEM, *P* values calculated by a two-tailed unpaired Student’s *t*-test. *P < 0.05, **P < 0.01, ***P < 0.001

To confirm the above findings, we sought to examine the effect of Fer-1, a ferroptosis inhibitor, on neointima formation after carotid artery ligation in mice in vivo (Fig. 3A). We confirmed that ferroptosis was inhibited after Fer-1 treatment through Perls’ Prussian blue staining and immunofluorescence analysis of COX-2 (Fig. 3B, C). Importantly, at 30 days after carotid artery ligation, H&E staining showed that Fer-1 treatment significantly decreased the intima-to-media ratio of injured carotid arteries (Fig. 3D). Furthermore, immunofluorescence staining showed that Fer-1 treatment markedly increased the expression of contractile protein α-SMA in neointima (Fig. 3E). Together, these data suggest that inhibition of ferroptosis attenuates neointimal hyperplasia in response to injury in mice in vivo.

### RSL3 promotes primary rat VSMC phenotypic switching

**Fig. 4:**
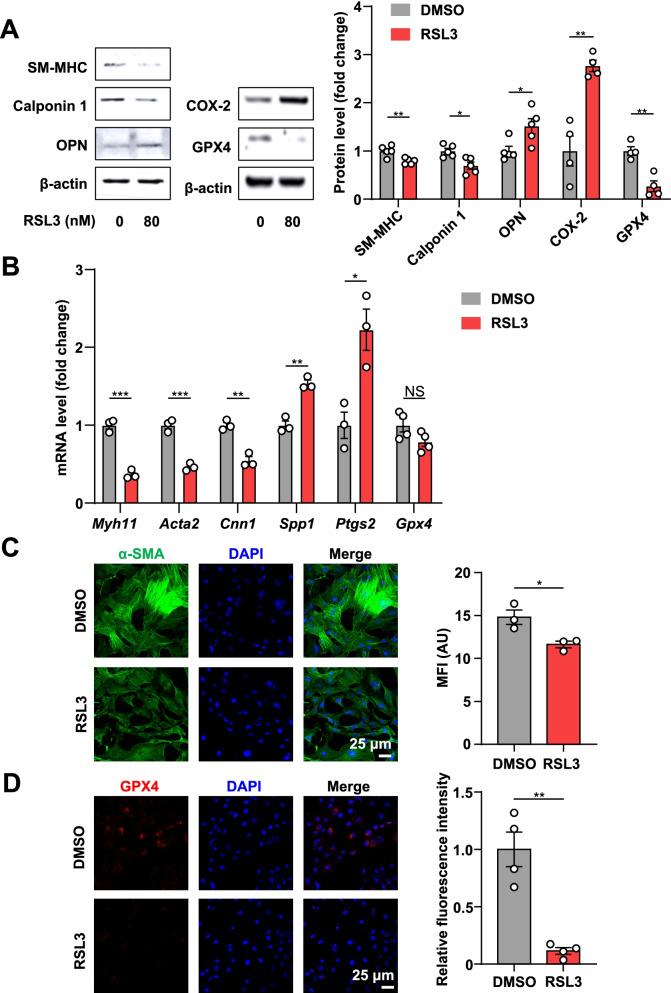
Ferroptosis inducer RSL3 promotes rat VSMC phenotypic switching. **A**–**D** Rat VSMCs were treated with or without RSL3 (80 nM) for 24 h, followed by further analysis. **A** The representative Western blots and densitometric quantification of SM-MHC, calponin 1, OPN, COX-2, and GPX4 (*n* = 4–5). **B** qRT-PCR analysis of *Myh11*, *Acta2*, *Cnn1*, *Spp1*, *Ptgs2* and *Gpx4* (*n* = 3–4). **C**, **D** The representative images of α-SMA and GPX4 and quantification of α-SMA MFI and GPX4 relative fluorescence intensity (*n* = 3). Data are presented as mean ± SEM, *P* values calculated by a two-tailed unpaired Student’s *t*-test. *P < 0.05, **P < 0.01, ***P < 0.001

To detect the effect of ferroptosis on the phenotypic switching of VSMCs, we isolated primary rat VSMCs from male SD rats and stimulated them with RSL3. As shown in Fig. 4A, RSL3 treatment significantly increased expression of COX-2 but significantly decreased expression of GPX4, indicating that ferroptosis was successfully induced in VSMCs. In line with in vivo data, RSL3 resulted in a significant decrease in the protein levels of contractile markers, including SM-MHC and calponin 1, but a marked increase in OPN, the synthetic marker, in rat VSMCs (Fig. 4A). Additionally, the mRNA levels of contractile markers, including *Myh11*, *Acta2*, and *Cnn1* decreased; whereas, the expression of synthetic marker *Spp1* and ferroptosis marker *Ptgs2* were increased (Fig. 4B). However, RSL3 treatment did not change *Gpx4* mRNA levels (Fig. 4B). We also found that the expression of α-SMA and GPX4 was decreased in rat VSMCs after RSL3 treatment (Fig. 4C, D). Collectively, these results show that induction of ferroptosis promotes rat VSMC phenotypic transition from the contractile to the synthetic phenotype.

### Fer-1 reverses RSL3-induced rat VSMC phenotypic switching

**Fig. 5:**
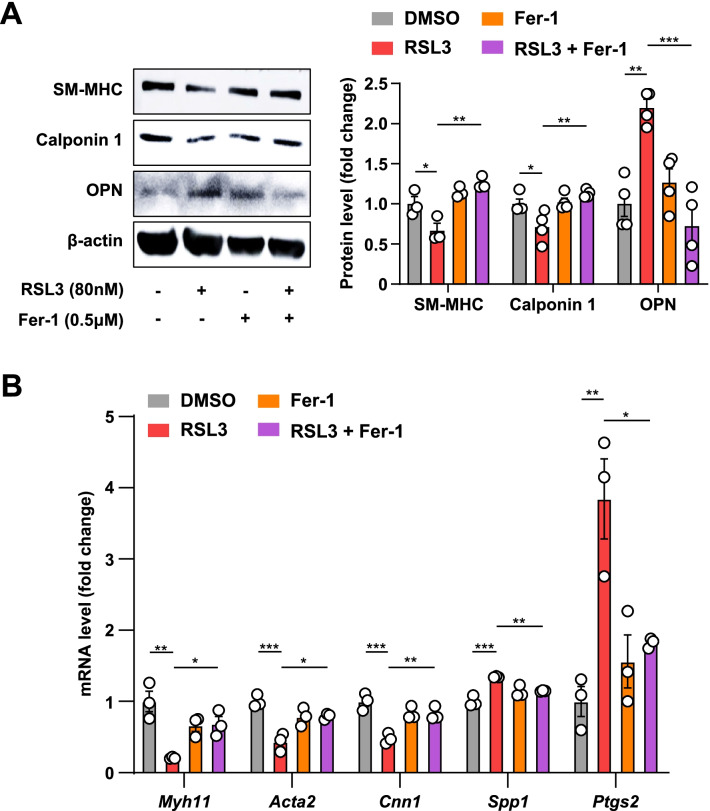
Ferroptosis inhibitor Fer-1 reverses RSL3-induced rat VSMC phenotypic switching. **A**, **B** Rat VSMCs were pretreated with Fer-1 (0.5 μM) for 15 min and/or RSL3 (80 nM) for an additional 24 h and subjected to the following analyses. **A** The representative Western blots and densitometric quantification of SM-MHC, calponin 1, and OPN (*n* = 3 and 4, respectively). **B** qRT-PCR analysis of *Myh11*, *Acta2*, *Cnn1*, *Spp1*, and *Ptgs2* (*n* = 3). Data are presented as mean ± SEM, *P* values calculated by a one-way ANOVA followed by Tukey’s multiple comparisons test. *P < 0.05, **P < 0.01, ***P < 0.001

To further confirm the contribution of ferroptosis to VSMC phenotypic switching, we asked if blocking of ferroptosis could abrogate RSL3-induced rat VSMC phenotypic transition. Rat VSMCs were treated with DMSO or Fer-1 in the presence or absence of RSL3. Consistent with the above results, RSL3 significantly downregulated the expression of contractile proteins (SM-MHC, calponin 1) but upregulated the expression of OPN (Fig. 5A). Importantly, Fer-1 rescued RSL3-induced downregulation of contractile proteins and inhibited upregulation of OPN (Fig. 5A). The results of mRNA analysis verified Western blot data (Fig. 5B). These results support that ferroptosis is actively involved in VSMC phenotypic conversion.

### ROS scavenger, NAC, negates RSL3-induced rat VSMC phenotypic conversion

**Fig. 6:**
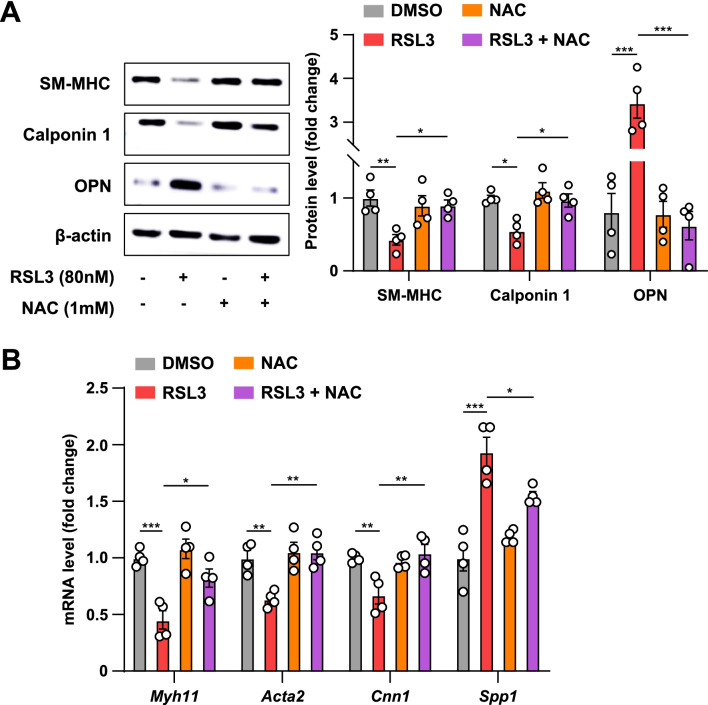
ROS scavenger NAC abolishes RSL3-induced rat VSMC phenotypic switching. **A**, **B** Rat VSMCs were pretreated with NAC (1 mM) for 15 min and/or RSL3 (80 nM) for an additional 24 h and subjected to the following analyses. **A** The representative Western blots and densitometric quantification of SM-MHC, calponin 1, and OPN (*n* = 4). **B** qRT-PCR analysis of *Myh11*, *Acta2*, *Cnn1*, and *Spp1* (*n* = 4). Data are presented as mean ± SEM, *P* values calculated by a one-way ANOVA followed by Tukey’s multiple comparisons test. *P < 0.05, **P < 0.01, ***P < 0.001

Eventually, we asked how ferroptosis affects rat VSMC phenotypic switching. On the one hand, excessive ROS and free iron lead to ferroptosis. On the other hand, redox signaling is an essential regulator of VSMC phenotypic switching (Vendrov et al. 2019). ROS is a critical member of these redox signaling pathways and contributes to VSMC phenotypic switching (Lu et al. 2018). Thus, we hypothesized that ferroptosis induced VSMC phenotypic conversion in a ROS-dependent manner. To this end, we incubated rat VSMCs with NAC, a well-known free radical scavenger, in the presence or absence of RSL3. Western blots showed that RSL3 downregulated the expression of contractile proteins (SM-MHC and calponin 1) and upregulated the expression of OPN; however, NAC reversed these effects (Fig. 6A). Consistently, qRT-PCR analysis showed that NAC reversed the effects of RSL3 on rat VSMC phenotypic switching (Fig. 6B). These data indicate that the effect of ferroptosis on rat VSMC phenotypic transition requires ROS.

## Discussion

**Fig. 7:**
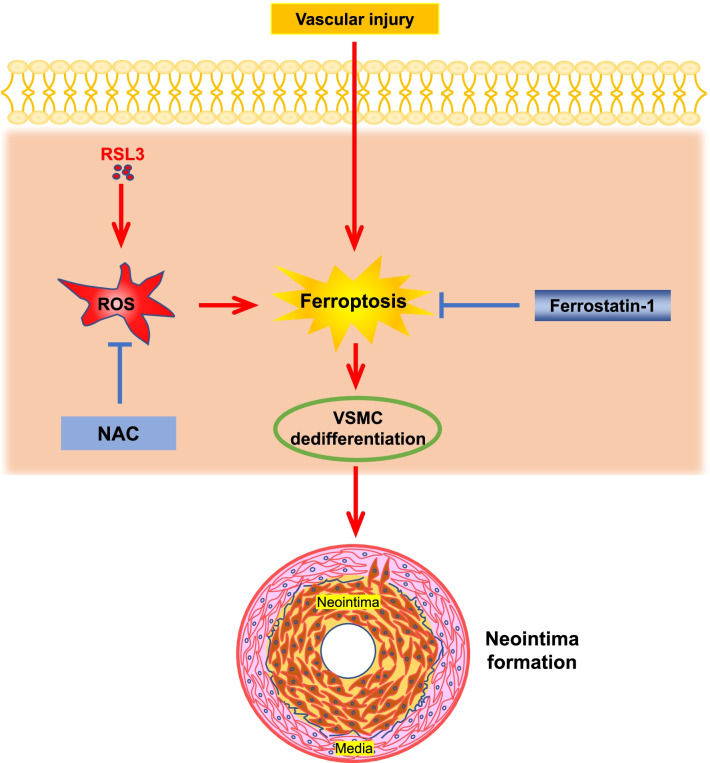
A schematic model showing that ferroptosis promotes VSMC phenotypic switching in a ROS-dependent manner and consequently aggravates mouse ligation-induced neointimal formation of carotid arteries

Ferroptosis is associated with a variety of cardiovascular diseases, such as ischemia/reperfusion-induced cardiomyopathy, doxorubicin-induced cardiotoxicity, heart failure, stroke, and aortic dissection (Li et al. 2021). However, the roles of ferroptosis in VSMC phenotypic switching and neointima formation remain unknown. By using various in vivo and in vitro models, we unveiled a previously uncharacterized role of ferroptosis in VSMC phenotypic switching and neointima formation (Fig. 7). Specifically, we have revealed that ferroptosis occurs during neointima formation. In the carotid artery ligation model, we found that induction of ferroptosis aggravated neointimal hyperplasia, while inhibition of ferroptosis reduced it. We observed that induction of ferroptosis triggered VMSC phenotypic switching, which was abrogated by a ferroptosis inhibitor. Moreover, we uncovered that ROS mediates ferroptosis-induced VMSC phenotypic switching. Thus, our results strongly indicate that ferroptosis plays a crucial role in neointima formation.

Previously, Zhou et al. showed that expression of Ptgs2 and ACSL4 were upregulated in atherosclerosis, while GPX4 was down-regulated in atherosclerosis, confirming ferroptosis in human coronary atherosclerosis (Zhou et al. 2021). Furthermore, the severity of atherosclerosis was positively correlated with the expression of Ptgs2 and ACSL4 and negatively correlated with the expression of GPX4 (Zhou et al. 2021). A recent study demonstrated that inhibition of ferroptosis relieves atherosclerosis by reducing lipid peroxidation and endothelial dysfunction in mouse aortic endothelial cells (Bai et al. 2020). Consistently, GPX4 overexpression in *ApoE*^*−/−*^ mice was shown to protect against atherosclerosis (Guo et al. 2008). Conversely, iron overload is one crucial characteristic of ferroptosis and was reported to promote atherosclerosis (Vinchi et al. 2020). In this study, we found that ferroptosis was induced during injury-induced vascular neointima formation. We also observed a positive correlation between ferroptosis and vascular neointimal formation.

Above all, using a common right carotid artery ligation model in mice in vivo we have shown that induction of ferroptosis significantly accelerated neointima formation; whereas, inhibition of ferroptosis limited neointima formation. After vascular injury, VMSCs undergo phenotypic modulation from a static contractile to an active synthetic phenotype and migrate from media to intima. VSMCs proliferate rapidly, synthesize and secrete extracellular matrix, resulting in neointima formation and vascular stenosis (Jackson 1994; Clowes et al. 1989). Sampilvanjil et al. (2020) revealed that cigarette smoke extract (CSE) induces ferroptosis in VSMCs, which may lead to aortic aneurysms and dissections. CSE significantly promoted cell death of rat VSMCs, which was not reversed by inhibitors of apoptosis or necroptosis but was reversed by Fer-1, liproxstatin-1, and iron chelator (Sampilvanjil et al. 2020). Fer-1 partially reversed the fragmentation and injury of mitochondria in the media of the aorta exposed to CSE (Sampilvanjil et al. 2020). We discovered that Fer-1 treatment significantly neutralized RSL3-induced VSMC phenotypic switching, identifying an essential role of ferroptosis in promoting VSMC switching from a contractile phenotype to a synthetic phenotype.

Another crucial finding of this study is that ferroptosis-induced neointimal hyperplasia is ROS-dependent. One main characteristic of ferroptosis is the imbalance between the generation and degradation of ROS. The accumulation of ROS increases lipid peroxidation, which eventually leads to cell death. RSL3 is a small molecule compound that can directly bind to and inhibit the activity of GPX4, leading to blocked ROS elimination and triggering of ferroptosis (Yang et al. 2014). ROS levels and transferrin expression were elevated in RSL3-treated colorectal cancer cells, while GPX4 expression was reduced (Sui et al. 2018). Cellular ROS and labile iron increase ferroptosis in a dose- and time-dependent manner (Sui et al. 2018). Oxidative stress is closely related to the pathophysiology of cardiovascular disease, including hypertension, atherosclerosis, aortic aneurysm, and vascular restenosis (Chan and Chan 2014; Kattoor et al. 2017; Irace et al. 2021; Vendrov et al. 2006). ROS is a regulator of VSMC phenotypic switching (Clempus and Griendling 2006). Numerous studies have shown that ROS induces the dedifferentiated phenotype of VSMC, thereby promoting VSMC proliferation (Griendling and Ushio-Fukai 1998). ROS can promote the dedifferentiation, proliferation, and migration of VSMCs through the NF-κB/mTOR/P70S6K pathway (Lu et al. 2018). *Ptgs2* encodes COX-2 and is a central gene in ferroptosis (Zhu et al. 2021). We found that RSL3 treatment significantly upregulated the expression of *Ptgs2* in VSMCs, indicating the occurrence of ferroptosis. RSL3-induced ferroptosis increases the accumulation of intracellular ROS. Therefore, we speculate that RSL3 promotes VSMC phenotypic switching through ROS. To test this, we used the ROS scavenger NAC. Our results showed that NAC counteracted VSMC phenotypic switching caused by RSL3.

We have demonstrated the role of ferroptosis in promoting neointima formation, but the underlying molecular mechanism reminds unclear. First, how does ferroptosis enable VSMC phenotypic switching from a contractile to a synthetic phenotype through ROS in vitro? Second, does ferroptosis promote neointima formation by increasing VSMC proliferation and migration? Furthermore, we do not know the effect of local adventitial administration of Fer-1 on neointimal hyperplasia of carotid arteries, which can avoid unknown non-specific or toxic effects related to systemic administration of the drug.

## Conclusions

Our results suggest that ferroptosis promotes neointima formation after arterial injury in mice and VMSC phenotypic transition from a differentiated contractile to a dedifferentiated synthetic phenotype. Ferroptosis-induced VMSC phenotypic switching is associated with ROS accumulation. Thus, our findings support that inhibition of ferroptosis or limiting ROS generation is a promising strategy to treat occlusive vascular diseases caused by restenosis of arteries following surgical interventions and injury.

## Data Availability

The datasets used and/or analyzed during the current study are available from the corresponding author on reasonable request.

## Abbreviations

VSMC: Vascular smooth muscle cell
α-SMA: α-Smooth muscle actin
SM-MHC: Smooth muscle myosin heavy chain
OPN: Osteopontin
ROS: Reactive oxygen species
RSL3: RAS-selective lethal 3
NAC: *N*-Acetyl-l-cysteine
Fer-1: Ferrostatin-1
COX-2: Cyclooxygenase-2
